# Hybrid equivalent circuit-deep neural network design of optically transparent metasurface with microwave RCS reduction

**DOI:** 10.1016/j.isci.2026.115212

**Published:** 2026-03-04

**Authors:** Liming Si, Tianyu Ma, Lin Dong, Rong Niu, Chenyang Dang, Xiue Bao, Kaiqiang Zhu, Houjun Sun, Weiren Zhu

**Affiliations:** 1 School of Integrated Circuits and Electronics, Beijing Key Laboratory of Millimeter Wave and Terahertz Technology, State Key Laboratory of Environment Characteristics and Effects for Near-Space, Beijing Institute of Technology, Beijing 100081, China; 2 State Key Laboratory of Radio Frequency, Heterogeneous Integration and Department of Electronic Engineering, Shanghai Jiao Tong University, Shanghai 200240, China

**Keywords:** physics, applied sciences, devices

## Abstract

Metasurface-based ultra-wideband radar cross-section (RCS) reduction techniques that offer flexibility and optical transparency are strategically important for electromagnetic protection. Although many methods, including machine learning techniques, have been developed to design complex metasurfaces, they typically require prior knowledge and extensive computational resources. Herein, we propose a physics-guided intelligent design approach to develop flexible optically transparent metasurfaces, which integrates the circuit analog optimization method (CAOM) with a deep neural network (DNN). As a proof-of-concept, a flexible, optically transparent metasurface for ultra-wideband RCS reduction was designed, fabricated, and experimentally characterized. The experimental results align closely with the simulations, demonstrating excellent flexibility and wide-angle RCS reduction from 8.9 to 37.2 GHz (123% fractional bandwidth). The metasurfaces exhibit excellent optical transparency with a visible transmittance of approximately 75%. The proposed metasurface shows great potential for integration into the window glass of stealth aircraft and warships, as well as solar-powered vehicles.

## Highlights

• Physics-guided intelligent design combining circuit-analog optimization with DNN proposed
• Flexible and optically transparent metasurface developed for ultra-wideband RCS reduction
• Achieves ≤−10 dB mono/bistatic RCS reduction across 8.9–37.2 GHz (123% bandwidth)
Fabricated sample exhibits ∼75% visible transmittance and excellent angular stability

## Graphical abstract

**Figure:**
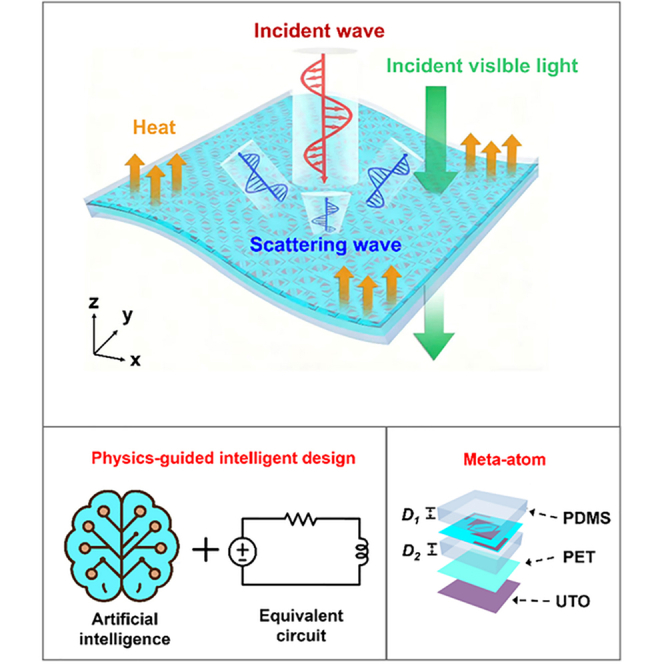

## Introduction

Metasurfaces composed of periodic or aperiodic subwavelength elements have attracted extensive interest because they are ultra-thin, lightweight, and offer highly tailorable electromagnetic (EM) responses.^1^^,^^2^^,^^3^ By appropriately engineering the unit-cell geometry and its spatial arrangement, they can provide full control over polarization, phase, and amplitude of EM waves, thereby enabling functionalities such as anomalous refraction/reflection, polarization conversion, and vortex-beam generation within a unified framework.^4^^,^^5^^,^^6^ At the same time, the rapid growth of wireless communication and digital electronic systems has heightened concerns about EM radiation exposure and EM compatibility, which further underscores the need for compact, high-performance EM control surfaces. The continued advancement of radar detection technologies has enhanced the early detection capabilities of aircraft and sensitive electronic equipment, making stealth and anti-detection demands increasingly critical.^7^ Driven by these factors, metasurfaces have become a research hotspot in both fundamental studies and applied engineering and are being progressively integrated into the EM shaping and scattering engineering of complex platforms. In stealth performance evaluation, the radar cross-section (RCS) is a key metric for assessing target detectability, which can be used to determine target presence and localization.^8^ Consequently, metasurface-based, thin-profile structures offer a unified platform for EM functionality modulation and scattering suppression and have received increasing attention in the fields of stealth and EM protection.

Metasurface-based RCS reduction technologies have effectively addressed the integration challenges posed by bulky absorbers and frequency selective surfaces on curved or conformal platforms^9^ and have been widely adopted in various related designs.^10^^,^^11^^,^^12^^,^^13^^,^^14^ The underlying mechanisms that have been explored include chessboard configurations for phase cancellation,^15^ in-phase reflection paths inspired by artificial magnetic conductors (AMCs),^16^ and polarization conversion metasurfaces (PCMs).^17^^,^^18^ However, these approaches are often constrained by limited bandwidth, angular sensitivity, or relatively large overall thickness. To overcome these limitations and achieve ultra-wideband RCS reduction of 10 dB or more, hybrid metasurfaces that integrate multiple mechanisms have been proposed.^19^ Nevertheless, most existing designs still fall short in meeting the flexibility and optical transparency requirements of curved targets or wearable devices. As a result, microwave stealth technologies that offer flexibility, optical transparency, and programmability have drawn considerable attention.^20^^,^^21^^,^^22^ Although transparent conductive oxides (TCOs) and graphene have been widely employed to construct optically transparent metasurfaces due to their dual capability in visible transparency and radio-frequency/microwave functionality, TCOs suffer from high sheet resistance and mechanical brittleness upon bending, while graphene often requires multilayered structures, metallic grids, or complex transfer processes to reduce resistance. These factors introduce additional loss, increase design complexity, and limit mechanical flexibility.^23^^,^^24^^,^^25^^,^^26^^,^^27^^,^^28^ Therefore, developing metasurfaces that simultaneously exhibit ultra-wideband performance, flexibility, and transparency remains an urgent need for next-generation stealth platforms.

Conventional broadband RCS reducing metasurface design primarily relies on design experience, parameter sweeps, and full-wave EM simulations. Although reported methodologies such as equivalent-circuit modeling, characteristic mode analysis, and Jones-matrix based approaches can reduce computational costs by providing simplified surrogates, they are generally inadequate for capturing complex, highly heterogeneous structures.^29^^,^^30^^,^^31^ As the geometric and material complexity increases, the associated computational burden grows rapidly. To address these challenges, deep neural networks (DNNs) have been employed to accelerate the design process by learning nonlinear mappings between geometric features and EM responses.^32^^,^^33^^,^^34^ Despite their strong predictive capability, DNNs typically require large, well-labeled datasets, offer limited physical insight, and often generalize poorly beyond the training domain. For example, introducing additional dielectric layers or geometric variations can invalidate a pretrained model, necessitating costly retraining.^35^^,^^36^^,^^37^^,^^38^ These limitations underscore the need to couple physically interpretable analytical models with the representational power of neural networks. Embedding physical priors or analytical constraints into DNN architectures can improve efficiency, interpretability, and generalization, thereby offering a more balanced framework for intelligent metasurface design.

In this work, we propose a physics-guided intelligent design method that combines the circuit analog optimization method (CAOM) with machine learning to develop a flexible and optically transparent metasurface for RCS reduction. The technical novelty lies in using CAOM to generate physics-guided and physically plausible absorption-conversion ratio (ACR) targets and coupling them with a closed-loop, CAOM-PSO-DNN design workflow, which reduces reliance on blind parameter sweeps while improving efficiency and physical interpretability by predicting circuit-derived performance metrics, rather than employing a purely black-box surrogate. This hybrid framework integrates equivalent circuit analysis—by extracting resistance (*R*), inductance (*L*), and capacitance (*C*) parameters of the surface unit—with data-driven prediction to accelerate optimization. A flexible and optically transparent metasurface prototype was designed and fabricated, employing a hybrid mechanism that combines polarization conversion and absorption within a checkerboard configuration to enhance scattering suppression. The metasurface achieves monostatic and bistatic RCS reduction of at least 10 dB across 8.9–37.2 GHz (123% fractional bandwidth) and demonstrates stable wide-angle and conformal performance. The fabricated sample also exhibits excellent optical transparency (∼75%) and mechanical flexibility, validating the proposed physics data-driven design strategy and offering a promising route toward the rapid and efficient development of wearable or conformal EM stealth devices.

## Results

### Physics-guided intelligent design framework

**Figure 1:**
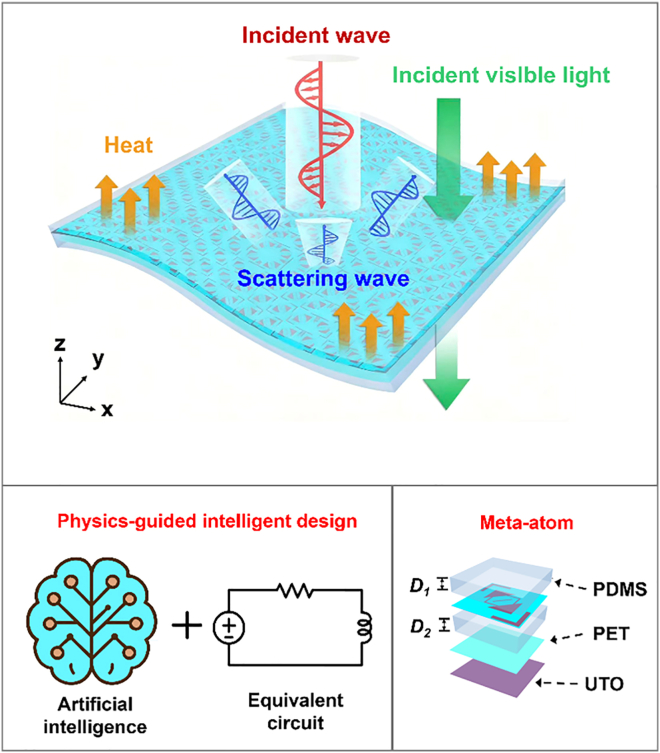
Schematic of the flexible optically transparent metasurface designed using physics-guided intelligent design method

**Figure 2:**
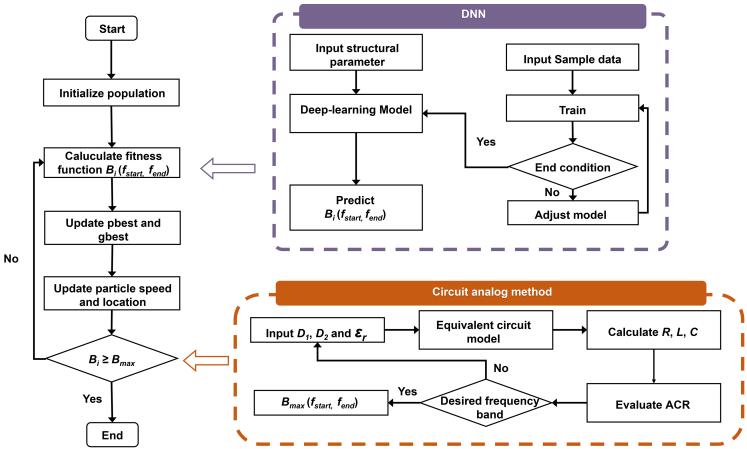
The flowchart of physics-guided intelligent design method

Figure 1 schematically illustrates the physics-guided intelligent design method, which comprises three components—the CAOM,^39^ particle swarm optimization (PSO), and a DNN. Specifically, Figure 2 presents the flowchart of the proposed method. The CAOM is employed to calculate the theoretical ACR, which serves as a key metric for the PSO algorithm. Moreover, the “physics-guided” insight is primarily extracted at the CAOM stage by establishing the dielectric-metal-dielectric-metal equivalent circuit, from which the effective *R*, *L*, and *C* parameters and the impedance-matching condition can be explicitly derived. This circuit-level interpretation provides a physical rationale for the broadband response (e.g., dual-resonance behavior) and is further used to define physically plausible ACR targets and bandwidth objectives for the subsequent optimization.

During the optimization process, the PSO evaluates the fitness of the ACR curves, updates particle velocities and population positions at each iteration, and iteratively proposes candidate structures for EM evaluation, which provide the training samples used to train the DNN. The DNN model is constructed together with the proposed metasurface structure, which is characterized by eleven structural parameters. These parameters constitute the input layer of DNN. The trained DNN model, comprising the input layer, several hidden layers, and a dropout layer, predicts the start and end frequencies of the continuous ACR with bandwidth exceeding 90% based on the structural parameters. These predictions are then fed back into the PSO to evaluate the fitness of the ACR curves and determine the final optimal parameters, thereby enabling an efficient closed-loop search guided by CAOM-derived physical criteria, rather than blind parameter sweeps. In this way, the DNN serves as a fast surrogate for bandwidth evaluation during optimization, while the CAOM-derived targets provide physics-based criteria for the PSO loop; therefore, the optimization is guided by circuit-based physical priors, rather than a purely black-box exploration.

The proposed pattern and the bottom structures of the metasurface are fabricated from indium tin oxide (ITO), with a sheet resistance of 6 Ω and a thickness of 185 nm. Although the thickness of the bottom ITO layer (185 nm) is smaller than the calculated skin depth, its sheet resistance (∼6 Ω/sq) is much lower than the free-space impedance (377 Ω), resulting in negligible transmission in the microwave band. Polyethylene terephthalate (PET, *ε*_*r*_ = 3.05, tan*δ* = 0.003) is used as the substrate for the ITO, but it is not considered a dielectric layer in the equivalent circuit model because of its extremely small thickness.

Polydimethylsiloxane (PDMS, *ε*_*r*_ = 2.7, tan*δ* = 0.06) serves as the top and middle dielectric material. Here, we establish an equivalent circuit for the four-layer unit structure (excluding the PET layers), as shown in Figure 1. Compared with the conventional sandwich structure, the four-layer design incorporates an additional dielectric layer, which can be represented in the equivalent circuit model to achieve broadband absorption while also protecting the top ITO layer. The top ITO structure is designed to be anisotropic, enabling efficient absorption and polarization conversion of incident EM waves. Moreover, no transmission occurs due to the presence of the metallic ITO ground; therefore, the absorption of the metasurface is determined solely by the reflection coefficient *R*(*ω*), which includes both co- and cross-polarized components. Typically, the absorption can be expressed as:

(Equation 1) $$A\left(\omega \right)=1-|S_{11}^{xx}\left(\omega \right){|}^{2}-|S_{11}^{yx}\left(\omega \right){|}^{2}=1-\frac{{\left[\left(Re\left(Z_{in}\right)-Z_{0}\;\cos\;\theta \right)\right]}^{2}+{\left[Im\left(Z_{in}\right)\right]}^{2}}{{\left[\left(Re\left(Z_{in}\right)+Z_{0}\;\cos\;\theta \right)\right]}^{2}+{\left[Im\left(Z_{in}\right)\right]}^{2}}$$

where $S_{11}^{xx}\left(\omega \right)$ and $S_{11}^{yx}\left(\omega \right)$ are the co-polarized and cross-polarized reflection coefficients, respectively, *Z*_in_ is the input impedance of the metasurface, *Z*_0_ = 377 Ω is the free space characteristic impedance, and *θ* is the incident angle of the EM wave. The absorption is affected by the input impedance of the equivalent circuit according to Equation 1. The equivalent circuit of the metasurface can be constructed by the transmission line theory, as shown in Figure 1. Based on the CAOM, the top ITO structure is equivalent to the series inductance *L*, capacitance *C* and resistance *R*, while the dielectric layers are treated as transmission line with characteristic impedance *Z*_n_. Due to the extremely small thickness of the PET layers, they can be negligible. Therefore, for the dielectric layers, only PDMS layers are considered. The characteristic impedance can be expressed as follows:

(Equation 2) $$Z_{a}=R_{g}$$

(Equation 3) $$Z_{1}=Z_{2}=\frac{Z_{0}}{\sqrt{\varepsilon r\left(PDMS\right)}}$$

(Equation 4) $$Z_{b}=Z_{1}\frac{Z_{a}+jZ_{1}\;\tan\left(\beta_{1}D_{1}\right)}{Z_{1}+jZ_{a}\;\tan\left(\beta_{1}D_{1}\right)}$$

where $\beta_{1}=2\pi f\sqrt{\varepsilon_{r}\left(\text{PDMS}\right)}/c$ represents the propagation constant, *f* is the frequency of incident waves, and *c* is the speed of light. Additionally, *D*_1_ and *D*_2_ are the thicknesses of the two PDMS layers. Because the function of the bottom ITO layer is the same as that of a metal ground, *R*_g_ can be approximated as zero. Therefore, *Z*_b_ can be expressed as follows:

(Equation 5) $$Z_{b}=j\frac{Z_{0}}{\sqrt{\varepsilon r\left(PDMS\right)}}\tan\left(\beta_{1}D_{1}\right)$$

while the impedance of the top ITO structure could be written as:

(Equation 6) $$Z_{RLC}=R+j\left(2\pi fL-\frac{1}{2\pi fC}\right)$$

Then, the input impedance *Z*_in_ can be expressed as:

(Equation 7) $$Z_{in}=\frac{\frac{Z_{0}\;\tan\left(\beta_{1}D_{1}\right)Z_{RLC}}{Z_{0}\;\tan\left(\beta_{1}D_{1}\right)-j\sqrt{\varepsilon r\left(PDMS\right)}Z_{RLC}}+\frac{Z0}{\sqrt{\varepsilon r\left(PDMS\right)}}\tan\left(\beta_{1}D_{2}\right)}{1+\frac{jZ_{0}\sqrt{\varepsilon r\left(PDMS\right)}\tan^{2}\left(\beta 1D1\right)Z_{RLC}}{Z_{0}\;\tan^{2}\left(\beta 1D1\right)-j\sqrt{\varepsilon r\left(PDMS\right)}Z_{RLC}}}$$

Based on *Z*_in_ = *Z*_0_, two equivalent circuit relationships of the metasurface can be obtained as follows:

(Equation 8) $$R=\frac{Z_{0}\;\tan^{2}\left(\beta D_{1}\right)\tan\left(\beta d_{2}\right)+Z_{0}\;\tan^{2}\left(\beta D_{1}\right)}{\varepsilon r{\left(1-\tan\left(\beta D_{1}\right)\tan\left(\beta D_{2}\right)\right)}^{2}+{\left(\tan\left(\beta D_{1}\right)+\tan\left(\beta D_{2}\right)\right)}^{2}}$$

(Equation 9) $$2\pi fL=\frac{z_{0}\;\tan\left(\beta D_{1}\right)\left(1-\tan\left(\beta D_{1}\right)\tan\left(\beta D_{2}\right)\right)}{\sqrt{\varepsilon r}\left(1-\tan\left(\beta D_{1}\right)\tan\left(\beta D_{2}\right)\right)+{\left(\tan\left(\beta D_{1}\right)+\tan\left(\beta D_{2}\right)\right)}^{2}}-\frac{z_{0}\;\tan\left(\beta D_{1}\right)\tan\left(\beta D_{2}\right)\left(\tan\left(\beta D_{1}\right)+\tan\left(\beta D_{2}\right)\right)}{{\varepsilon r}^{\frac{3}{2}}{\left(1-\tan\left(\beta D_{1}\right)\tan\left(\beta D_{2}\right)\right)}^{2}+\sqrt{\varepsilon r}{\left(\tan\left(\beta D_{1}\right)+\tan\left(\beta D_{2}\right)\right)}^{2}}+\frac{1}{2\pi fC}$$

It can be seen from Equation 1 that the absorption of the metasurface can be determined by the selected dielectric materials from Equation 7, Equation 8, and Equation 9. For polarization conversion and absorption, the ACR is usually applied to describe the performance of the hybrid mechanism. Meanwhile, to better analyze the polarization-conversion and absorption capabilities of this type of metasurface, the ACR is commonly used to evaluate its control over x-polarized EM waves. The expression for ACR is as follows:

(Equation 10) $$ACR=1-|S_{11}^{xx}\left(\omega \right){|}^{2}$$

The polarization-conversion capability of this type of metasurface is evaluated by the cross-polarized reflection ratio:

(Equation 11) $$C\left(\omega \right)=|S_{11}^{yx}\left(\omega \right){|}^{2}$$

### Circuit-based analysis and ACR-oriented optimization

**Figure 3:**
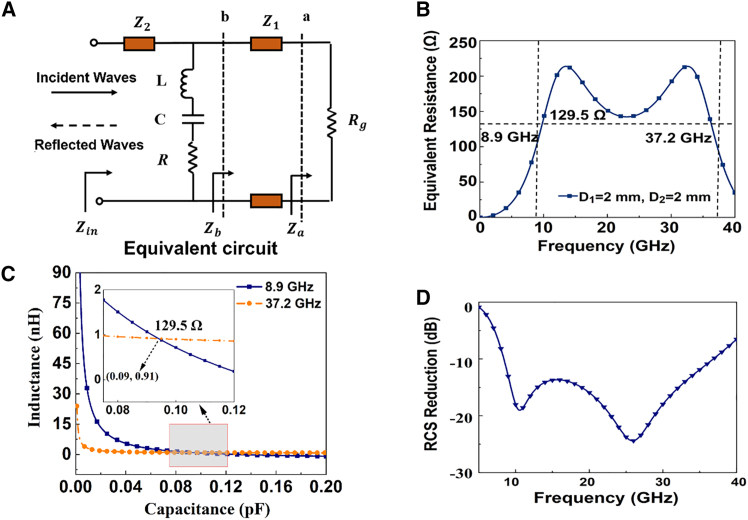
Equivalent circuit schematic and theoretical calculation value (A) Equivalent circuit. (B) Equivalent resistance (*D*_1_ = *D*_2_ = 2 mm). (C) The equivalent inductance *L* versus the equivalent capacitance *C*. (D) Theoretical RCS reduction value.

The analysis and theoretical results corresponding to Figures 3A–3D are presented as follows. Figure 3A illustrates the equivalent circuit model of the designed unit cell, which forms the basis for deriving the equivalent parameters. Figure 3B shows the frequency-dependent equivalent resistance when *D*_1_ and *D*_2_ are both set to 2 mm, calculated using Equation 8. On this basis, Figure 3C depicts the relationship between the equivalent inductance *L* and the equivalent capacitance *C* obtained from Equation 2, where their intersections determine the feasible parameter combinations that satisfy impedance matching. Finally, Figure 3D presents the theoretical RCS reduction corresponding to the selected equivalent parameters. Among these, the case with *L* = 0.09 nH and *C* = 0.91 pF yields the optimal absorption response, which meets the desired operating frequency range of 8–40 GHz and is, therefore, chosen as the target absorption curve.

Current research indicates that an accurate relationship can be established between equivalent circuit parameters and simple surface structures. However, establishing such a relationship for complex structures is challenging and often limited to qualitative analysis. Unfortunately, such analysis lacks sufficient accuracy. To address this issue, we incorporate theoretical calculation results into the PSO and DNN algorithms to optimize the structure, thereby replacing the qualitative analysis of complex structures. This method also reduces the number of parameters and the size of the training dataset related to the dielectric layer. Although, in theory, both absorption and polarization conversion may be affected by structural changes, the resistive material provides an equivalent resistance *R*, which makes the impact of structural variations on absorption stronger than on polarization conversion. Thus, the fitness absorption curve is defined as the fitness ACR curve in the PSO and DNN optimization algorithms. Specifically, the equivalent resistance *R* primarily governs the absorption level and bandwidth, while the coupled *L*-*C* combinations determine the impedance-matched resonance locations; this provides an interpretable circuit-level guideline for the subsequent PSO-DNN optimization.

To achieve an RCS reduction of −10 dB, more than 90% of the incident EM energy must be either absorbed or converted through polarization. Therefore, the PSO and DNN algorithms are employed to optimize the unit cell, where the start and stop frequencies of the absorption coefficient region (ACR) with *τ* ≥ 90% are defined as the fitness function:

(Equation 12) $$\left\{ \begin{array}{c} Range\left(X\right)=\left(f_{start}\left(X\right),f_{end}\left(X\right)\right),{\;B}_{\max}\left(X\right)=f_{end}\left(X\right)-f_{start}\left(X\right)\;\\ \max\;B_{\max}\left(X\right),X\in \Delta \; \end{array}\right.$$

where Range(*X*) returns the start and end of the longest continuous interval within [*f*_min_, *f*_max_], ACR(*f*;X)≥*τ. X* = [*r*_1_, *r*_2_, *r*_3_, *r*_4_, *r*_5_, *L*_1_, *c*_1_, *c*_2_, *p*_1_, *p*_2_, *p*_3_, *p*] ^T^ is the vector of the structural parameters of the top ITO layer, as shown in Figure 2B, and *Δ* is the parameter space. *B(X)* is the maximum continuous bandwidth of ACR exceeding 90%. In the optimization, the start and stop frequencies (*f*_start_(*X*), *f*_end_(*X*)) that define *B(X)* are fed into the PSO and DNN models as the fitness/targets.

The top ITO structure can be obtained by applying the boolean operations of the eleven-dimensional parameters detailed in Table 1. In addition, the DNN training process has three steps. Firstly, we call the CST Microwave Studio from PSO to adjust values of the eleven parameters and modify the metasurface structure; many training samples are generated during this process. Secondly, we design the DNN model, including an input layer, several hidden layers, and an output layer. The input layer consists of the eleven parameters, while the output layer is Range(*X*). All the parameters and frequency points are normalized to enhance the training accuracy. The hidden layers consist of three fully connected layers and one dropout layer. The fully connected layers have 50, 50, and 20 neurons, while the dropout layer deactivates some neurons to improve the model accuracy and robustness. The DNN topology (three hidden layers with 50, 50, and 20 neurons) was selected to balance prediction performance and model complexity for the low-dimensional tabular dataset (11 input parameters, ∼6000 samples), thereby reducing overfitting risk while maintaining sufficient representational capacity. Leaky ReLU is adopted as the activation function to provides nonlinearity, which is expressed as:

(Equation 13) $$LeakyReLU\left(x\right)=\left\{ \begin{array}{c} x,x>0\\ \alpha x,x\leq 0 \end{array}\right.$$

Here, Leaky ReLU can solve the zero-gradient defect of the negative value, and α is close to 0.01. The mean square error (MSE) function is adopted as the loss function:

(Equation 14) $$MSE=\frac{1}{n}\sum_{i=1}^{n}{\left(Y_{i}-\hat{Y_{i}}\right)}^{2}$$

where *n* is the number of samples, *Y*_*i*_ and $\hat{Y_{i}}$ are the actual and predicted values, respectively. Thirdly, we randomly select 5,500 samples produced from the first step as the training dataset and another 500 samples as the testing dataset. Although the inputs are normalized, the hidden-layer pre-activations can still become negative during training. Therefore, Leaky ReLU is adopted to improve optimization stability and mitigate the ‘dying ReLU’ issue by preserving a small non-zero gradient in the negative region, i.e., *f*(*x*) = *x* for *x* ≥ 0 and *f*(*x*) = *ax* for *x* < 0, where *a* = 0.01 in this work.

**Table 1:** The range of each dimensional parameters and final values

Parameters	Range (mm)	Final parameters (mm)
*r*^*a*^	4.00–10.00	4.63
*r*^*b*^	0.20–6.00	5.65
*r*^*c*^	0.10–8.20	8.00
*r*^*d*^	0.10–8.00	5.70
*r*^*e*^	0.10–3.30	0.34
*L*^*a*^	0.10–3.00	2.94
*c*^*a*^	0.10–7.00	3.00
*c*^*b*^	0.10–3.20	0.20
*p*^*a*^	7.0–10.0	9.6
*p*^*b*^	0.10–3.00	0.55
*p*^*c*^	0.10–8.00	6.12
*p*	–	10.00 mergeindicator:codebox_paragrpah:end

**Figure 4:**
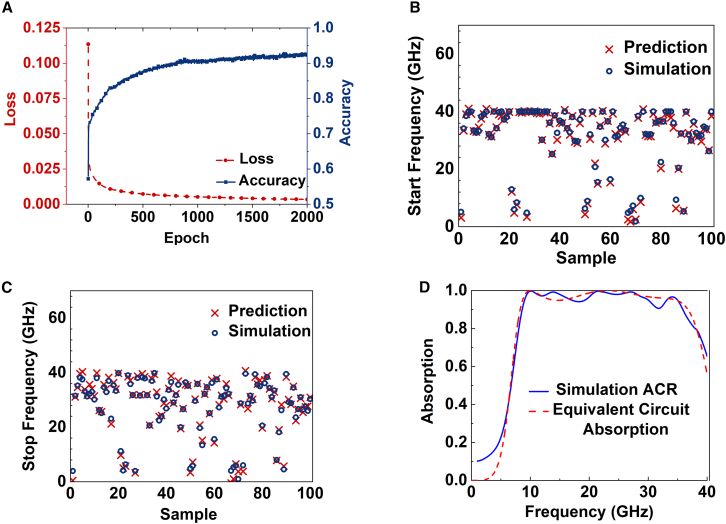
DNN training data and comparison between theoretically calculated ACR and simulated ACR data (A) The accuracy and loss of the training process. (B and C) The predicted frequency points (start and stop) and simulated frequency points of 100 samples randomly selected from test sets. (D) The comparison of simulation ACR and equivalent circuit absorption.

As shown in Figure 4A, the training process of the DNN model converges steadily after 2,000 epochs, with the loss decreasing to below 0.0003. A tolerance-based prediction accuracy is defined as the percentage of samples for which the absolute difference between the predicted value and the ground-truth value is no greater than 0.1. Based on this criterion, a tolerance-based accuracy of approximately 92% is achieved. To quantitatively assess the regression performance, the trained DNN achieves a root-mean-square error (RMSE) of 5.2364 × 10^−7^, a mean absolute error (MAE) of 4.875 × 10^−4^, and an R^2^ score of 0.96589 on the test dataset, indicating high prediction fidelity and good generalization capability. Figures 4B and 4C compare the randomly predicted start and stop frequency points corresponding to ACR >90% obtained from the trained DNN module with those from full-wave simulations. The predicted and simulated results show good agreement, demonstrating that the trained DNN can effectively predict the operating frequency range. Furthermore, Figure 4D presents the simulated and calculated ACR responses, which are consistent with each other and satisfy the design objectives.

The DNN delivers the best overall regression performance, while the random forest is a competitive and more interpretable, lower-cost baseline typical for small tabular datasets. The support vector regression (SVR) performs poorly under the current setup, indicating limited fit to this dataset. These results support using the DNN when accuracy is the priority, with random forest as a practical alternative when efficiency and interpretability matter most. These data are presented in Table 2.

**Table 2:** Test performance comparison for DNN, SVR, and RF models

Model	RMSE	MAE	R^2^
DNN	5.2364E-07	0.000488	0.96589
SVR	2.39901E-05	0.00431	−0.41661
RF	1.01085E-06	0.000713	0.936029 mergeindicator:codebox_paragrpah:end

### Checkerboard super-cell design and RCS reduction mechanism

**Figure 5:**
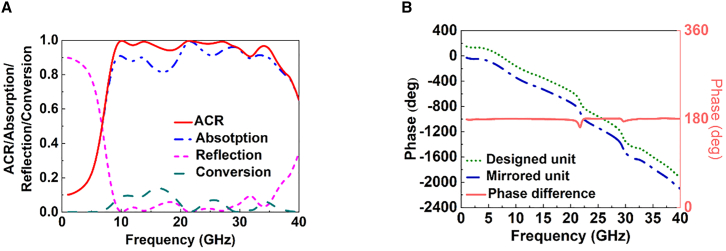
Contributions of absorption and polarization conversion to ACR and the comparison of phases (A) The comparison of the ACR, absorption, conversion, and reflection. (B) The results of reflection phase and phase difference.

The final optimized parameters are shown in the second row of Table 1. Because a single unit cell cannot reduce the RCS via the phase-cancellation principle, a 4 × 4 super-cell is proposed. This super-cell, consisting of 4 × 4 elements, represents either “0” or “1” and simulates periodic boundary conditions. Moreover, Figure 5A shows the absorption and polarization conversion contributions to the ACR, which indicates that the average absorption and polarization conversion are over 85% and 8% from 8.64 to 35.91 GHz, respectively. In addition, the average co-polarization reflection rate is less than 5%. The polarization conversion ratio (*PCR*) characterizes the polarization conversion efficiency, which can be expressed as:

(Equation 15) $$PCR=\frac{|S_{11}^{yx}\left(\omega \right){|}^{2}}{|S_{11}^{yx}\left(\omega \right){|}^{2}+|S_{11}^{xx}\left(\omega \right){|}^{2}}$$

The metasurface achieves an average *PCR* exceeding 80% across 9.46–16.71 GHz and 24.94–27.60 GHz, consistent with the corresponding conversion peaks within the operating band. This result indicates that the metasurface efficiently converts the residual co-polarized incident wave into the cross-polarized component after absorption. By leveraging this capability, a 180° phase difference can be introduced using a mirrored structure, realized by rotating the designed structure by 90°. The phase differences are illustrated in Figure 5B. The reflection phase difference remains close to 180° across the entire operating frequency range, indicating that the metasurface can achieve RCS reduction via the phase-cancellation principle.

The metasurface comprising the proposed unit cell and its mirrored structure can be used to achieve RCS reduction. The far-field scattering pattern of the whole M × N array can be written as:

(Equation 16) $$SP\left(\theta ,\varphi \right)=\cos\;\theta \sum_{m=1,n=1}^{M,N}e^{-j[\left(\frac{2\pi }{\lambda }\sin\;\theta \right)\left(\cos\;\varphi \cdot mp_{x}+\sin\;\varphi \cdot np_{y}\right)-\varphi_{mn}]}$$

where *θ* and *φ* represent the elevation and azimuth angles, respectively. The integers *m* and *n* indicate unit cells along *x* and *y* directions with spacings *p*_*x*_ and *p*_*y*_; thus, the cell coordinates are *x*_m_ = *mp*_*x*_ and *y*_*n*_ = *np*_*y*_. *φ*_*mn*_ represents the scattering phase of the (*m*, *n*)^th^ unit cell. Due to the phase difference between the designed structure and mirrored structure, 1-bit coding metasurface can be constructed. Assuming an equal number of “0” and “1” units, the RCS reduction of the metasurface can be expressed as:

(Equation 17) $$RCS_{reduction}=10{\mathrm{lg}\left|\frac{SP{\left(\theta_{s},\varphi_{s}\right)}_{ms}}{SP{\left(\theta_{s},\varphi_{s}\right)}_{\text{metal}}}\right|}^{2}=10{\mathrm{lg}\left|\frac{A_{1}e^{j\varphi_{1}}+A_{2}e^{j\varphi_{2}}}{2}\right|}^{2}=10{\mathrm{lg}\left|\frac{A_{1}+A_{2}e^{j\Delta \varphi }}{2}\right|}^{2}$$

where *A*_1_ (*A*_2_) and *φ*_1_ (*φ*_2_) are the reflection amplitude and phase of the “0” (“1”) units, respectively, while Δ*φ* is the phase difference. (θ_s_, *φ*_*s*_) = (*π*-*θ*^*i*^, *φ*^*i*^*+π*). According to Equation 16 and Equation 17, the amplitude and phase differences mainly determine the RCS reduction, while the configuration of the elements controls the spatial scattering patterns of the metasurface.

**Figure 6:**
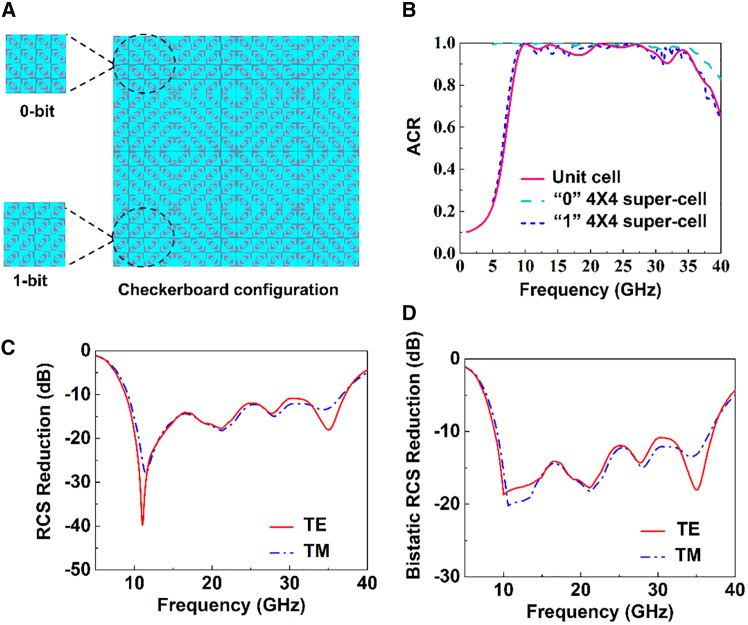
Chessboard-structured array, comparison of unit structures and 0-, 1-bit unit ACR, and TE/TM monostatic/bistatic reduction of the chessboard-structured 0-, 1-bit unit cells, with demonstration of the contributions of absorption and polarization conversion to ACR and the comparison of phases (A) The checkerboard configuration of the metasurface. (B) Comparison of the ACR for the unit cell and the 4 × 4 1-bit and 0-bit super-cells. (C and D) The result of the (C) monostatic and (D) bistatic RCS reduction with TE and TM modes of the checkerboard configuration of the metasurface.

Figure 6A displays the designed metasurface configuration that adopts a checkerboard pattern composed of the super-cell with 4 × 4 elements to represent “0” or “1” and mimic periodic boundary conditions. Besides, Figure 6B shows the performance of the 4 × 4 super-cells, which are almost the same as the unit cell. Additionally, a metallic plate with the same size is used as a reference to demonstrate the scattering suppression of the metasurface. Figures 6C and 6D show the monostatic and bistatic RCS reductions of the checkerboard configuration under different incident polarizations, demonstrating ≤ −10 dB monostatic and bistatic RCS reduction across 8.9–37.2 GHz. Moreover, Figures 6C and 6D indicate that the metasurface is nearly polarization insensitive. Therefore, the checkerboard configuration is adopted to realize ultra-wideband RCS reduction through a hybrid mechanism.

**Figure 7:**
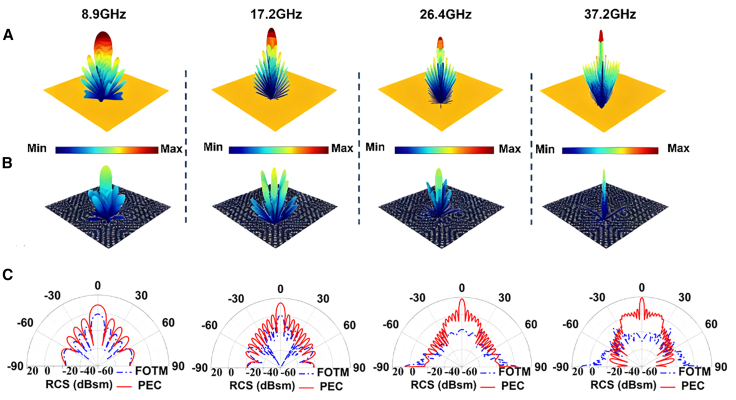
3D scattering patterns of the perfect conductor and checkerboard-configured FOTM, and their 2D backward scattering patterns at different frequencies (A) 3D scattering patterns of the perfect conductor. (B) 3D scattering patterns of the checkerboard-configured FOTM. (C) 2D backward scattering patterns of PEC and the checkerboard-configured FOTM at different frequencies. FOTM, flexible optically transparent metasurface; PEC, perfect electric conductor.

To further validate the RCS reduction performance of the checkerboard-configured metasurface, the 3D scattering patterns of the perfect electric conductor (PEC) and the metasurface at 8.9, 17.2, 26.4, and 37.2 GHz are depicted in Figures 7A and 7B, respectively. The results show that the proposed metasurface produces four main scattering beams under normal incidence, similar to conventional checkerboard metasurfaces. However, unlike traditional metasurfaces, the proposed design can absorb and convert reflected EM waves without satisfying the perfect scattering-cancellation condition. In addition, Figure 7C presents the 2D scattering patterns of the PEC and the metasurface at the same frequencies. It can be observed from Figure 7C that the proposed metasurface effectively suppresses the four main scattered beams, resulting in better RCS reduction performances.

### Experimental validation and performance evaluation

**Figure 8:**
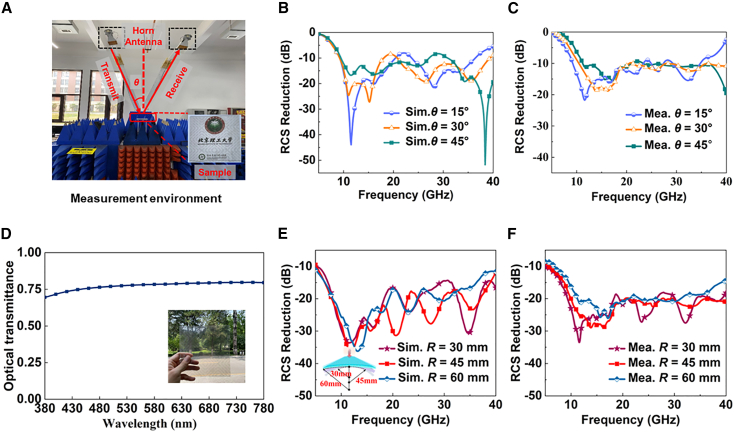
Experimental scenario, and comparison between the simulation and experimental data (A) Photographs of the measurement environment and the fabricated sample. (B) The results of the monostatic RCS reduction of FOTM with different θ. (C) Comparison of monostatic RCS-calibrated measurements and simulation results for a metasurface wrapped around cylinders of different radii. (D) Test results of visible light transmittance. (E) The results of FOTM wrapped on cylinders with different radii. (F) The monostatic RCS reduction measurements of FOTM wrapped on cylinders with different radii. FOTM, flexible optically transparent metasurface.

The proposed metasurface is fabricated by the microelectromechanical system technology to validate the full-wave simulations, and the fabricated sample is shown in Figure 8A. The Naval Research Laboratory (NRL) arch method is used to measure the metasurface, which consists of two pairs of standard broadband horn antennas and a vector network analyzer (Agilent N5224A), shown in Figure 8A as well. The operating frequency bands of the two pairs of antennas are from 2 to 18 GHz and 18 to 40 GHz. The measured RCS reduction results at different incident angles are shown in Figure 8C, and they are generally consistent with the simulated trends in Figure 8B. It can be seen from Figure 8C that the metasurface maintains a stable RCS reduction of at least 10 dB as the incident angle increases, validating its wide-angle RCS reduction capability. Moreover, Figure 8D shows the optical transparency of the fabricated sample; the visible transmittance is approximately 75% based on a photometer measurement under representative visible illumination. In addition, the measured RCS reduction results of the metasurface wrapped around cylinders with different radii are presented in Figure 8F. They are largely consistent with the simulated results in Figure 8E, thereby verifying the flexible RCS reduction capability. Specifically, as the bending radius decreases (i.e., the curvature increases), the curvature of the metasurface modifies the local reflection directions of the unit cells, causing the scattered EM energy to deviate from the specular reflection direction. This redistribution of scattering paths effectively suppresses the forward-scattered (monostatic) component, leading to a reduced RCS level across the operating band and thus an enhanced stealth performance. The minor discrepancies observed can be attributed to fabrication tolerances and measurement uncertainties.

To clarify the advantages of the proposed metasurface, Table 3 shows a comparison with recent similar works. Due to the rapid accurate design in global optimizations, the proposed metasurface features a wider relative bandwidth, a faster design process, flexible RCS reduction, and angular stability. Compared with most reported works, the 10 dB monostatic RCS reduction bandwidth of the proposed metasurface exceeds those in recent similar works summarized in Table 3, except for that in the work by Xi et al.^42^ However, the bistatic RCS reduction bandwidth in Xi et al.’s work^42^ is lower than that of the proposed metasurface, and it is not flexible. Besides, the proposed design method can effectively tackle the requirements of plenty of priori knowledge, optimization time, or computational resources in the traditional optimization methods. In addition, the metasurface can adopt different relative permittivity or thickness of dielectric substrates for application in different operating frequencies or environmental requirements.

**Table 3:** Comparison between the RCS reduction metamaterials reported in the literature and this study

Reference	10 dB monostatic RCS reduction bandwidth	10 dB bistatic RCS reduction bandwidth	Angular stability	Flexibility	Transparency	Mechanism	Design method
Fu et al.^27^	8.52–16.98 GHz/66.35%	–	<55°	yes	yes	absorption	design experience + sweep parameters
Zhang et al.^29^	7.00–18.00 GHz/92.30%	–	none	yes	yes	absorption	design experience + sweep parameters
Chen et al.^40^	8.00–15.00 GHz/60.86%	–	<40°	yes	yes	phase cancellation	design experience + sweep parameters
Lu et al.^17^	7.40–22.60 GHz /101.00%	7.40–22.50 GHz /100%	∼45°	no	no	polarization conversion	design experience + PSO
Ji et al.^41^	13.00–31.50 GHz /84.00%	–	–	no	no	hybrid mechanism	design experience + sweep parameters
Feng et al.^8^	12.37–28.44 GHz /77.80%	–	–	no	no	hybrid mechanism	design experience + sweep parameters
Xi et al.^42^	7.49–32.23 GHz/124.50%	7.46–30.55 GHz/121.50%	<45°	no	yes	hybrid mechanism	design experience + sweep parameters
This work	8.86–37.17 GHz/123.00%	8.86–37.13 GHz/123.00%	∼45°	yes	yes/75%	hybrid mechanism	CAOM + DNN mergeindicator:codebox_paragrpah:end

## Discussion

In this work, we propose a physics-guided intelligent design method that integrates the CAOM with machine learning to develop a metasurface capable of ultra-wideband RCS reduction. Specifically, a robust correlation is established between the structural parameters and the ACR based on equivalent transmission line/circuit theory, which is further combined with a DNN to rapidly predict the ACR of metasurfaces with different structural parameters and is subsequently embedded into a PSO loop for efficient optimization. For verification, a metasurface with a hybrid mechanism for RCS reduction was designed, fabricated, and experimentally characterized. The experimental results demonstrate that the proposed metasurface achieves monostatic and bistatic RCS reduction of at least 10 dB, from 8.9 to 37.2 GHz, together with an average optical transmittance of approximately 75% in the visible region. Furthermore, wideband scattering suppression is maintained for incident angles up to 45° and for cylindrical conformal configurations with radii as small as 30 mm, validating the excellent angular stability and mechanical flexibility and suggesting potential extensions toward wearable EM stealth devices.

Despite these promising results, several limitations and practical challenges remain to be addressed in future work. From a modeling perspective, the equivalent-circuit model is established under simplified assumptions (e.g., local periodicity and near-normal incidence), which may not fully capture strong coupling effects or complex scattering behaviors under highly oblique incidence; moreover, the DNN surrogate depends on the quality and coverage of the training dataset, and its black-box nature limits direct interpretability, while PSO may exhibit reduced convergence efficiency in higher-dimensional design spaces. From an engineering standpoint, real-world deployment on platforms such as stealth aircraft, naval vessels, and solar-powered vehicles will require further investigation of environmental durability (e.g., ITO oxidation, humidity/temperature cycling, and UV/moisture aging of PET/PDMS), interfacial reliability under repeated deformation, and scalable large-area manufacturing with uniform thickness, pattern fidelity, and stable electrical performance (e.g., roll-to-roll processing), as well as system-level integration compatibility with radomes/structural materials and long-term EM/mechanical robustness. Importantly, the proposed CAOM-DNN-PSO framework is inherently scalable beyond ultra-wideband RCS reduction; by redefining the fitness functions and EM targets, the same pipeline can be extended to multifunctional metasurface design for beam shaping, scattering redistribution, and angle/polarization-selective sensing, and it can be further adapted to future tunable or reconfigurable metasurfaces by incorporating bias-dependent material parameters or active elements into the physics-guided surrogate model.

### Limitations of the study

The present study still faces several limitations. The equivalent circuit model is established under simplified assumptions of local periodicity and normal incidence, which may not fully account for strong coupling effects or complex oblique incidence conditions. The DNN depends on the quality and coverage of training data, and its black-box nature limits the interpretability of the optimization process. Moreover, the PSO algorithm may encounter convergence inefficiency in high-dimensional parameter spaces. From an experimental perspective, the fabrication and measurement of flexible transparent metasurfaces remain technically challenging, and their stability under deformation and environmental variations requires further validation. Overall, while the proposed method achieves remarkable bandwidth and flexible RCS reduction, its scalability to multifunctional or reconfigurable metasurfaces still needs to be explored in future work.

## Resource availability

### Lead contact

Further information and requests for resources and reagents should be directed to and will be fulfilled by the lead contact, Liming Si (lms@bit.edu.cn).

### Materials availability

This study did not generate new reagents.

### Data and code availability

• All data reported in this paper will be shared by the lead contact upon request.
• Any additional information required to reanalyze the data reported in this paper is available from the lead contact upon request.

## Acknowledgments

This work was supported by the National Key RD Program of China (grant no. 2022YFF0604801), the 10.13039/501100001809National Natural Science Foundation of China (grant nos. 62271056, 62571049, 62171186, and 62201037), the Technology Innovation Center of Infrared Remote Sensing Metrology Technology of State Administration for Market Regulation (grant no. AKYKF2423), the Beijing Natural Science Foundation of China-Haidian Original Innovation Joint Fund (grant no. L222042), the 10.13039/501100003787Hebei Natural Science Foundation (grant no. F2025105029), the Open Research Fund of State Key Laboratory of Millimeter Waves (grant no. KN20250214), the Open Research Fund of State Key Laboratory of Space-Ground Integrated Information Technology (grant no. 6142221200201), the 10.13039/501100012398Basic Research Foundation of Beijing Institute of Technology, China (grant no. BITBLR2020014), and the 10.13039/501100013314111 Project of China (grant no. B14010). (Corresponding author: Liming Si.).

## Author contributions

Conceptualization, L.S. and W.Z.; methodology, T.M., R.N., and L.D.; software and simulations, T.M. and C.D.; investigation and data curation, T.M., L.D., R.N., X.B., and K.Z.; validation, L.D., X.B., and H.S.; writing – original draft, T.M. and R.N.; writing – review & editing, L.S. and W.Z.; resources, L.S. and W.Z.; funding acquisition, L.S. and W.Z.; supervision, L.S. and W.Z. All authors discussed the results and approved the final manuscript.

## Declaration of interests

The authors declare no conflicts of interest.

## STAR★Methods

### Key resources table

**Table:** 

REAGENT or RESOURCE	SOURCE	IDENTIFIER
**Software and algorithms**		

Origin 2021	OriginLab	https://www.originlab.com
CST Studio Suite 2020	Dassault Syste`mes	https://www.3ds.com
PyTorch	Meta AI	https://www.pytorch.org

### Method details

#### Material composition and layer structure

The proposed metasurface was fabricated using indium tin oxide (ITO) as both the patterned top layer and the continuous bottom layer. The ITO film had a sheet resistance of 6 Ω/sq and a thickness of 185 nm. The substrate supporting the ITO layers was polyethylene terephthalate (PET) with a relative permittivity of *ε*_*r*_ = 3.05 and a loss tangent of tan*δ* = 0.003; however, due to its extremely small thickness, PET was not considered a dielectric layer in the equivalent circuit model. Polydimethylsiloxane (PDMS) was used as the intermediate and top dielectric layers, with *ε*_*r*_ = 2.7 and tan*δ* = 0.06. The full stack formed a four-layer metasurface design excluding PET, and the equivalent circuit model was constructed accordingly to extract RLC parameters.

The patterned ITO layer (nominal thickness: 185 nm) was deposited on a 0.05 mm-thick PET substrate by magnetron sputtering, with thickness controlled by calibrated deposition parameters. Standard thin-film characterization methods (e.g., thickness verification and sheet-resistance evaluation using a four-point probe) are commonly used to assess the film quality and electrical uniformity of sputtered ITO layers.^43^ The patterned ITO/PET film was subsequently bonded to the PDMS dielectric layer using a UV-curable adhesive. The PDMS thickness was controlled by a spacer-defined molding/casting process; thickness uniformity is typically quantified by multi-point measurements and can be reported using a normalized metric (standard deviation divided by mean thickness) as adopted in prior literature.^44^ In the present work, we report the nominal thicknesses used for fabrication, while a systematic thickness mapping across the entire sample area will be explored in future work. Finally, the fabricated samples were characterized using a standard NRL arch system to evaluate the RCS-reduction performance.

#### Software tools and simulation platform

The structure was designed and simulated using CST Microwave Studio 2020, and all optimization and neural network training tasks were conducted using Python (with PyTorch backend).

#### Equivalent circuit modeling

Based on the CAOM framework, the unit structure was modeled by extracting its effective resistance (*R*), inductance (*L*), and capacitance (*C*) values. This model provided a physically interpretable descriptor of the electromagnetic absorption behavior. To improve design efficiency and reduce dependency on brute-force parameter sweeps, a hybrid intelligent framework was constructed by embedding the circuit descriptors into a deep neural network (DNN) and a particle swarm optimization (PSO) algorithm.

#### Optimization objective and fitness function

The design objective was to achieve >90% absorption or polarization conversion, corresponding to ≤ -10 dB RCS reduction. The optimization fitness function was defined as the maximum continuous bandwidth B(X) over which the absorption cross-section ratio (*ACR*) exceeds *τ=*90%, expressed as:

$$\left\{ \begin{array}{c} \text{Range}\left(X\right)=\left(f_{\text{start}}\left(X\right),f_{\text{end}}\left(X\right)\right),{\;B}_{\max}\left(X\right)=f_{\text{end}}\left(X\right)-f_{\text{start}}\left(X\right)\\ \max\;B_{\max}\left(X\right),X\in \Delta \end{array}\right.$$

where Range(*X*) returns the start and end of the longest continuous interval within[*f*_min_, *f*_max_], *ACR*(*f* ;X)≥*τ*. *X* = [*r*_1_, *r*_2_, *r*_3_, *r*_4_, *r*_5_, *L*_1_, *c*_1_, *c*_2_, *p*_1_, *p*_2_, *p*_3_, *p*] ^T^ denotes the 11 structural parameters defining the top ITO pattern geometry. These parameters were optimized within a defined design space Δ.

#### Neural network architecture and training

The DNN was constructed with:

• Input layer: 11 geometric parameters
• Hidden layers: 3 fully connected layers with 50, 50, and 20 neurons respectively, followed by one dropout layer to prevent overfitting
• Activation function: Leaky ReLU with slope *α* ≈ 0.01 for negative input
• Output layer: Predicted Range(*X*)

All inputs and outputs were normalized prior to training. The mean squared error (*MSE*) was used as the loss function:

$$MSE=\frac{1}{n}\sum_{i=1}^{n}{\left(Y_{i}-\hat{Y_{i}}\right)}^{2}$$

The training process of the DNN model converges steadily after 2000 epochs, with the loss decreasing to below 0.0003. A tolerance-based prediction accuracy is defined as the percentage of samples for which the absolute difference between the predicted value and the ground-truth value is no greater than 0.1. Based on this criterion, a tolerance-based accuracy of approximately 92% is achieved.

To enable fast surrogate prediction for the low-dimensional tabular dataset, we employed a fully connected deep neural network (DNN) with an 11-dimensional input corresponding to the geometric parameter vector. The network comprises three fully connected hidden layers with 50, 50, and 20 neurons, followed by an output layer. This topology was selected to balance prediction performance and model complexity for the dataset size (∼6000 samples), thereby reducing overfitting risk while maintaining sufficient representational capacity. Leaky ReLU was used as the activation function in all hidden layers to improve optimization stability and mitigate the “dying ReLU” issue by preserving a small non-zero gradient in the negative-input region. To further enhance generalizability, a dropout layer (dropout rate = 0.2) was applied during training (and disabled during inference). The model was trained using the Adam optimizer with a learning rate of 1×10^-3^ to minimize the mean squared error (MSE) loss, with a batch size of 32 for up to 2000 epochs; early stopping was employed by monitoring the validation loss to prevent overfitting. All input features (geometric parameters) and output labels were normalized prior to training, and the dataset was split into training/validation/test subsets.

The DNN was trained in PyTorch on an Intel® Xeon® Gold 6246R (3.40 GHz) CPU and an NVIDIA® GeForce® GTX 3090 GPU, reaching convergence in 2000 epochs within 5 minutes, while RF and SVM required 2 minutes and 3 minutes, respectively, and the trained DNN delivers millisecond-level predictions once the data are prepared. An end-to-end runtime benchmark further shows that the PSO+DNN optimization finishes in 8.5 minutes, substantially faster than conventional full-wave simulation and parameter sweeping.

#### Particle swarm optimization integration

The PSO algorithm called CST simulations iteratively and used the trained DNN as a surrogate model for faster convergence. Boolean operations on the 11-parameter design vector were used to generate valid structural variants. After training, the DNN is embedded into the PSO-based optimization framework . In each generation, each particle represents an 11-parameter unit-cell candidate, which is fed into the pretrained DNN to predict the required electromagnetic quantities (e.g., complex reflection/transmission coefficients over the target band). These predictions are then used to compute the ACR response and extract the corresponding bandwidth *Bi*, which serves as the fitness value for updating particle velocities and positions. The iteration terminates once *Bi* matches the CAOM-derived target bandwidth *B*max; otherwise, the next generation proceeds. This closed-loop design significantly reduces reliance on time-consuming full-wave simulations during optimization.

#### Fabrication

The final optimized structure was fabricated using standard thin-film deposition and lithographic patterning techniques to implement the designed ITO patterns on the PET/PDMS stack. Care was taken to preserve optical transparency during patterning.

Algorithm 1 **Deep Neural Network Structure and Training Procedure**

Input:
Dataset $D={\left\{\left(x_{i},y_{i}\right)\right\}}_{i=1}^{M}$ (11 input parameters, 2 output targets);
Learning rate *η* = 10^−3^; Batch size *B* = 32; Dropout rate *p* = 0.2; Epochs *E* = 2000.
Output:
Trained DNN model weights *θ*^∗^.
1: Data Preprocessing:
2: Split *D* into training set *D*_train_ (80%) and testing set *D*_test_ (20%)
3: Normalize input features *x* in *D*_train_ and *D*_test_
4: Network Architecture Construction:
5: Initialize fully connected layers with weights *θ*:
6:  Layer 1: Input *R*^11^→ 50 neurons, Activation: ReLU
7:  Layer 2: Hidden 50 → 50 neurons, Activation: ReLU
8:  Layer 3: Hidden 50 → 20 neurons, Activation: ReLU
9:  Layer 4: Output 20 → 2 neurons, Dropout(*p*) applied
10: Define forward pass function *f*_*θ*_(***x***)
11: Training Loop (Backpropagation):
12: Initialize optimizer (Adam) with learning rate *η*
13: for epoch *e* = 1 to *E* do
14:  Shuffle *D*_train_
15:  for each batch *B*_j_ ⊂ *D*_train_ of size *B* do
16:  Forward pass:
17:  Predictions $\hat{y}\leftarrow f_{\theta }\left(x_{\text{batch}}\right)$
18:  Loss calculation (MSE):
19:  $L\left(\theta \right)\leftarrow \frac{1}{B}\sum_{k=1}^{B}{\left\|y_{k}-{\hat{y}}_{k}\right\|}^{2}$
20:  Backward pass:
21:  Compute gradients $\nabla_{\theta }L$
22:  Update weights $\theta \leftarrow \text{Adam}\left(\theta ,\nabla_{\theta }L,\eta \right)$
23:  end for
24: end for
25: return Trained model $f_{\theta^{*}}$

Algorithm 2 **PSO Optimization with DNN-Predicted Bandwidth Fitness**

Input:
Pretrained DNN model *M*;
Search space bounds *L*, *U* ∈ ℝ^11^;
Swarm size *N*; Max iterations *T*_max_; Inertia *w*; Coefficients *c*_1_, *c*_2_;
Absorption threshold *δ* = 0.9.
Output:
Optimized structure parameters *x*^∗^ and max bandwidth *F*^∗^.
1: Initialization:
2: for *i* = 1 to *N* do
3:  Initialize position *x*_i_ ∈ [*L*, *U*] randomly (11 parameters)
4:  Initialize velocity *v*_i_ = 0
5:  Evaluate initial fitness: *F*_i_ ← CalcFitness(*x*_i_)
6:  Update personal best: *p*_i_ ← *x*_i_, *F*_i_^best^ ← *F*_i_
7: end for
8: Update global best: *g* ← argmax(*F*_i_^best^), *F*^∗^ ← max(*F*_i_^best^)
9: Main Loop:
10:  for *t* = 1 to *T*_max_ do
11:  for *i* = 1 to *N* do
12:  //Update velocity and position
13:  *v*_i_ ← *w*·*v*_i_ + *c*_1_·*r*_1_·(*p*_i_ - *x*_i_) + *c*_2_·*r*_2_·(*g* - *x*_i_)
14:  *x*_i_ ← *x*_i_ + *v*_i_
15:  Clip *x*_i_ to bounds [*L*, *U*]
16:
17:  //Evaluate fitness using DNN
18:  *F*_i_ ← CalcFitness(*x*_i_)
19:
20:  //Update personal and global bests
21:  if *F*_i_ > *F*_i_^best^ then
22:  *p*_i_ ← *x*_i_, *F*_i_^best^ ← *F*_i_
23:  if *F*_i_ > *F*^∗^ then
24:  *g* ← *x*_i_, *F*^∗^ ← *F*_i_
25:  end if
26:  end if
27:  end for
28: end for
29: return *x*^∗^ ← *g*
30: Function CalcFitness(*x*)
31:  Predict absorption spectrum *A* ← *M*(*x*)
32:  Identify frequency range where *A* ≥ *δ*
33:  Calculate continuous bandwidth *BW*
34: return *BW*

### Quantification and statistical analysis

The simulation data are produced by CST Microwave Studio software. Figures shown in the main text were produced by Origin.

### Additional resources

Any additional information about the simulation and data reported in this paper is available from the lead contact on request.
